# Trends in Use of Alcohol and Cigarettes among Danish Adolescents, 2002–2018: Exclusive and Dual Use

**DOI:** 10.3390/ijerph19063490

**Published:** 2022-03-15

**Authors:** Simone G. Kjeld, Lisbeth Lund, Katrine R. Madsen, Mogens T. Damsgaard, Lotus S. Bast

**Affiliations:** National Institute of Public Health, University of Southern Denmark, Studiestraede 6, 1455 Copenhagen, Denmark; lilu@sdu.dk (L.L.); krma@sdu.dk (K.R.M.); trab@sdu.dk (M.T.D.); loni@sdu.dk (L.S.B.)

**Keywords:** alcohol, smoking, tobacco, cigarette, substance use, dual use, adolescents, trends

## Abstract

Many young adolescents experiment with substance use which can have substantial health implications later in life. This study examined trends in substance use among Danish adolescents from 2002 to 2018, including exclusive and dual current use of alcohol and cigarettes. Data on 13- and 15-year-olds (N = 15,295) from five comparable cross-sectional Health Behavior in School-aged Children (HBSC) surveys were used. Cochran-Armitage test for trend assessed the development in substance use patterns over time. Overall, a decreasing trend in current use of alcohol and cigarettes was found among Danish adolescents during the 16-year study period: from 71.7% in 2002 to 51.6% in 2018. In 2018, most adolescents (41.8%) currently used alcohol exclusively, 8.6% had a dual current use of cigarettes and alcohol, and 1.3% smoked cigarettes exclusively. Trends in alcohol use differed according to age groups, while no gender-specific trends in substance use were found. Findings suggest that a significant prevention potential in adolescent substance use remains, and future initiatives may focus on dual use of substances as well as tailored efforts to specific subgroups in high risk of using substances.

## 1. Introduction

During the last decades, smoking prevalences have decreased globally among the general population and also among adolescents–especially since the turn of the millennium [1,2]. However, recent Danish studies show somewhat equivocal trends; some results suggest an increase in smoking [1,3], while the latest measures indicate that adolescent cigarette smoking is again on a slowly decreasing path [4,5,6]. Previous research suggests that smoking uptake may differ according to gender, i.e., boys are more prone to smoke cigarettes compared with girls [7], although recent Danish measures suggests no notable differences in smoking prevalences across gender [8]. Moreover, smoking initiation and prevalence increases with increasing age [9].

Danish adolescents are known to have a high alcohol intake compared to adolescents in other European countries [10]. The proportion of Danish youth that currently consumes alcohol has been rather stable over the last decade [11]. Moreover, Danish 15-year-olds hold first place in binge drinking (intake of five or more alcoholic units in one sitting) compared with other European countries, although among 11- to 13-year-olds, the proportion who binge drinks is more on par with the European average [10]. Across most European countries, no pronounced gender-specific differences have been found in relation to alcohol use [8].

The high proportion of adolescents engaging in substance use (i.e., cigarettes and alcohol) is considered a critical public health issue [12,13]. Substance use is typically initiated in adolescence, and most individuals have initiated their use of alcohol and cigarettes before the age of 18 years [5,9,14]. Therefore, it is essential for preventive efforts to explore substance use among the youngest adolescents. The health hazards associated with substance use increase with earlier age of initiation, and the brain development is especially at-risk with early initiation of smoking and alcohol use [13,15], e.g., due to high levels of nicotine and other harmful chemicals, i.e., carcinogens, in conventional cigarettes. Further, initiating substance use in adolescence and youth predicts continued use in adulthood, heavier use, and difficulties with cessation [16,17]. One significant consideration is the clustering of risk behaviors, i.e., using one substance may increase the likelihood of trying or regularly using others [18]. In addition, simultaneous use of tobacco and alcohol is relevant to examine because of the social contexts in which they are often initiated as well as the social implications associated with engaging in these substance use behaviors, e.g., adolescents tend to perceive alcohol as a means of building and maintaining friendships with peers [19,20]. Mapping the trends in exclusive and dual substance use among young adolescents will help inform researchers and practitioners in preventive efforts that target adolescents’ risk behaviors. Moreover, knowledge about possible subgroup differences will help tailoring interventions to adolescents most in need of prevention efforts.

Therefore, the present study sought to examine trends in substance use among Danish adolescents (13- and 15-year-olds) since the turn of the millennium—from 2002 to 2018. More specifically, this study examined adolescents’ exclusive current use of cigarettes and alcohol, respectively, as well as dual current use of cigarettes and alcohol. Analyses were based on the total study population and divided into subgroups based on age, gender, as well as age and gender.

## 2. Materials and Methods

### 2.1. Study Design

The current study reports data from the Danish contribution to the international collaborative cross-national Health Behavior in School-aged Children (HBSC) study, which collects data from 11-, 13- and 15-year-olds adolescents every fourth year using an internationally standardized HBSC questionnaire that is completed in the classroom [21,22]. The HBSC study aims at enhancing the understanding of adolescents’ well-being and health behaviors in their social settings. Hence, the present study draws on repeated cross-sectional surveys of representative samples of Danish adolescents at five time points: 2002, 2006, 2010, 2014, and 2018, respectively. The sampling procedures, data collection, and measurements were similar across all included surveys. At each time point, schools were chosen at random, i.e., cluster sampling, from a complete list of private and public schools in Denmark. Each survey included all adolescents at the enrolled schools in grade 5, 7, and 9 (corresponding to 11-, 13-, and 15-year-olds) with mean ages (SD) of 11.4 (0.4), 13.4 (0.4) and 15.4 (0.4), respectively. The response rate (measured as the mean percentage of participating adolescents enrolled in the participating school classes across the five surveys) was 87.0%. In this study, the youngest age group (11-year-olds) was excluded for further analyses as the prevalence of substance use among this group is fairly low compared with older adolescents. Subsequently, the total number of included participants in this study was N = 15,295.

### 2.2. Measures

#### 2.2.1. Sociodemographic Characteristics

The following variables were reported for characterizing the study population at each time point: *gender*, *grade* (divided into grade 7 corresponding to approx. 13 years and grade 9 corresponding to approx. 15 years), *country background* (born in Denmark vs. other, i.e., immigrant or descendant of immigrants), and adolescents’ *family occupational social class (OSC)* measured by two questions about the occupation of adolescents’ father and mother. The highest-ranking parent determined the OSC which was categorized into three groups: high, medium, low, and non-classifiable comprised of adolescents who could not be categorized in the latter categories.

#### 2.2.2. Exclusive and Dual Current Use of Cigarettes and Alcohol

Current cigarette and alcohol use were assessed by adolescents’ responses to two items: (1) “How often do you smoke cigarettes?” dichotomized into ‘Do not smoke cigarettes’ vs. ‘Current use of cigarettes (‘Daily; ‘Weekly; ‘More seldom’), and (2) “At present, how often do you drink anything alcoholic like (a) beer; (b) wine; (c) spirits/liquor; or (d) alcopops” dichotomized into ‘Do not use alcohol’ vs. ‘Current use of alcohol’ (‘Every day’, ‘Every week’, ‘Every month’, or ‘Rarely’). Adolescents reporting either current use of cigarettes or alcohol were categorized into *any current use*, while adolescents who reported neither substance use behaviors were categorized into *neither using cigarettes nor alcohol*.

Further, *any current use* was categorized into *exclusive current use of cigarettes* comprised of adolescents that exclusively smoked cigarettes, *exclusive current use of alcohol* comprised of adolescents that exclusively used alcohol, and *dual current use of cigarettes and alcohol*.

### 2.3. Analyses

The 9.4 version of SAS was used for all statistical analyses. Descriptive analyses were conducted for adolescents’ sociodemographic and substance use characteristics stratified by survey year. Second, trends in substance use patterns were analyzed using Cochran-Armitage test for trend for the total population and stratified by gender, age as well as gender and age, respectively. A *p*-value of < 0.05 was used to assess statistical significance.

## 3. Results

### 3.1. Characteristics of Participating 13- and 15-Year-Olds Adolescents

Characteristics of the participating adolescents at each survey year are presented in Table 1. Almost half of adolescents were girls (50.8% in 2002 and 48.9% in 2018), and a little more than half were in grade 7 (corresponding to approx. age 13). Across all survey years, most adolescents had either a high or a medium OSC and were born in Denmark.

As shown in Table 2, very few smoked exclusively cigarettes (0.8% in 2002 versus 1.3% in 2018, *p* = 0.030). Exclusive current use of alcohol decreased from 52.8% in 2002 to 41.8% in 2018 (*p* < 0.001), and dual current use of cigarettes and alcohol decreased from 18.1% to 8.6% (*p* < 0.001).

### 3.2. Current Substance Use by Age and Gender

In Table 3, trends in current substance use were stratified by age groups (grade 7 and 9 corresponding to age 13 and age 15, respectively). Results showed that any current use declined among adolescents in grade 7, from 57.5% in 2002 to 30.7% in 2018 (*p* < 0.001). Similarly, for adolescents in grade 9, any current use declined from 87.6% to 78.7% (*p* < 0.001). Slightly more adolescents in grade 7 smoked exclusively in 2018 (1.6%) than in 2002 (0.9%). A significant decline in exclusive current alcohol use was found among adolescents in grade 7—from 46.0% in 2002 to 26.3% in 2018 (*p* < 0.001). Among adolescents in grade 9, no significant differences in exclusive current alcohol use were found (*p* = 0.493). For dual current use of cigarettes and alcohol, a declining trend was found among adolescents in grade 7 and grade 9—from 10.7% in 2002 to 2.8% in 2018 among adolescents in grade 7 (*p* < 0.001), and from 26.5% in 2002 to 16.1% in 2018 among adolescents in grade 9 (*p* < 0.001).

In Table 4, trends in current substance use were stratified by gender. Results showed that any current use declined among both genders over the study period—from 72.7% in 2002 to 54.0% in 2018 among boys (*p* < 0.001), and from 70.7% to 49.1% among girls (*p* < 0.001). Over time, there was a minor increase in exclusive current use of cigarettes among boys (from 0.5% in 2002 to 1.4% in 2018). Among both girls and boys, a decreasing trend in exclusive current alcohol use was found. Among boys, exclusive current alcohol use decreased from 54.4% in 2002 to 43.4% (*p* < 0.001), while among girls, the corresponding values were 51.2% and 40.1% (*p* < 0.001), respectively. For dual current use of cigarettes and alcohol, declining trends were detected among boys (from 17.9% in 2002 to 9.3% in 2018, *p* < 0.001) and among girls (from 17.9% in 2002 to 9.3% in 2018, *p* < 0.001).

In Table 5, trends in current substance use were stratified by gender *and* age groups. Any current use declined among all subgroups. Significant declines in exclusive current alcohol use among girls and boys in grade 7 were found (boys: from 49.0% in 2002 to 29.6% in 2018, *p* < 0.001, girls: from 43.0% in 2002 to 23.0% in 2018, *p* < 0.001). Among both genders in grade 9, there were no significant differences in exclusive current alcohol use (boys: *p* = 0.732, girls: *p* = 0.525). There were not enough observations in some of the cells to report trends in exclusive current use of cigarettes. Regarding dual current use of cigarettes and alcohol, decreasing trends were found across gender and age groups (boys in grade 7: from 11.6% to 3.4%, *p* < 0.001, boys in grade 9: from 25.2% to 16.8%, *p* < 0.001, girls in grade 7: from 9.8% to 2.2%, *p* < 0.001, girls in grade 9: from 25.2% to 16.8%, *p* < 0.001).

## 4. Discussion

This study examined trends in adolescents’ exclusive and dual current use of cigarettes and alcohol. Findings showed overall decreasing trends in current use of cigarettes and alcohol among Danish adolescents over the 16-year study period. These findings may indicate that national preventive efforts have contributed to an overall reduction in adolescent substance use. However, in 2018, still a little more than half of the adolescents currently used alcohol and about 9% also smoked cigarettes. Thus, a significant prevention potential in adolescent substance use behaviors remains. Understanding the development in substance use behaviors and how the engagement in substance use changes over time is essential for future prevention efforts. Specifically, this study showed that trends in substance use differed markedly according to age groups and less according to gender.

We found that the overall proportion of adolescents reporting to smoke cigarettes decreased over the study period; from about one in five adolescents in 2002 to about one in ten in 2018. Regulations on tobacco have developed markedly during the last two decades, and the Danish government introduced several smoking control policies that may have impacted smoking prevalences [23]: Smoking was banned among adolescents on the school premises in 2000, although teachers were still allowed to smoke. In 2004, the sale of tobacco products was prohibited to people younger than 16 years, and since 2008, this has applied to adolescents below the age of 18. Since 2012, there has been a complete ban on smoking on school premises. The decreasing trends in cigarette smoking along with legislative efforts may indicate the possible strong impacts of structural interventions. In the most recent Danish legislation on tobacco, several measures are initiated to prevent especially youth smoking, including tax increases, ban on added flavors such as fruit and mint, a ban of promotion at the point of sale, standardized packaging, health warnings on all products containing nicotine, increased age control, smoke-free school time, and an extended ban on advertising and sponsorship of tobacco and nicotine-containing products. However, these initiatives were not initiated at the time of the study. Future studies may investigate whether the recently adopted legislations on tobacco and nicotine use are linked with further decreased use of cigarettes. Moreover, future research may employ measures of other drug and substance use to clarify whether decreasing trends in cigarette smoking are replaced by corresponding increases in other substance use.

A very small proportion smoked cigarettes exclusively, and the minor increases in exclusive smoking found in this study, i.e., among boys and adolescents in grade 7, should be followed in the years to come to detect whether these are actual trends or merely due to random variations over time, e.g., differences in sampling procedures. Although few adolescents smoked cigarettes exclusively, a higher proportion of adolescents had a dual current use of cigarettes and alcohol. These findings indicate that cigarette smoking is very seldom initiated without initiating other risk behaviors such as alcohol. This is in line with previous research showing the development of alcohol use and smoking patterns are closely related, i.e., alcohol use has been shown to predict tobacco use and vice versa [24], which may to some extent be explained by the social settings in which substance use behaviors are initiated [19,20].

A large proportion of Danish 13- and 15-year-olds currently used alcohol (approx. 50%). These findings align with existing knowledge on Danish alcohol intake in adolescence, which shows that alcohol is an essential aspect of youth life [10]. Moreover, there was a stagnation in the development of current alcohol use from 2014 to 2018. Divided into subgroups, findings showed decreasing trends in alcohol use among both genders from 2002 to 2014, although a slightly larger share of boys used alcohol over the entire study period compared with girls. Other research in this area did not find gender-specific differences in alcohol use among adolescents in Denmark and other European countries [8,11], and the differences between boys and girls in this study were not markedly pronounced. A significant drop in current alcohol use was also found among adolescents in grade 7 over the study period, while among adolescents in grade 9, no significant downwards trend was found. In fact, almost the same proportion currently used alcohol in 2018 as in 2002 (more than 60%). These findings are in line with previous numbers on Danish 15-year-olds alcohol intake [11]. Divided into groups based on age *and* gender, the trends among boys and girls in grade 9 were similar, thus, indicating that the stagnation in alcohol use among older adolescents was not gender-specific. The causes of this stagnation should be further investigated, and newer and up-to-date measurements are needed to determine whether these trends will continue among adolescents.

Explanations of the continuing high alcohol use prevalence among Danish adolescents may include the lack of structural efforts aiming at adolescent alcohol use in Denmark compared with structural initiatives on, e.g., tobacco products. In 2004, the sale of alcohol was prohibited for adolescents younger than 16 years, and from 2011, adolescents younger than 18 years are not allowed to buy alcoholic beverages with an alcohol by volume (ABV) of 16,5% or more. In most other Scandinavian and European countries, there is a minimum age limit on buying any alcohol of 18 years [25]. Consequently, the moderate Danish structural initiatives may to some extent explain the higher rates of alcohol intake among Danish adolescents. Moreover, as the highest proportion in Europe, 95% of 15- to 16-year-old Danes perceive it as easy to access and acquire alcoholic beverages. The corresponding average for the other European countries is 78% [26]. Recently, however, there has been increasing focus on adolescent alcohol use and problematic outcomes of the current youth alcohol culture. For example, many adolescents (more than half of respondents) in Denmark had experienced ‘drinking pressure’, i.e., feeling pressured to drink more than they felt like [27]. New initiatives have accordingly been implemented to reduce alcohol use and drinking pressure, e.g., by introducing alcohol policies in the school arena [28].

The current study found that adolescent substance use is overall decreasing among 13- and 15-year-olds in Denmark, which should be considered a positive development. However, there is still a high proportion of adolescents currently using one or two substances—and especially alcohol remains popular among adolescents. Given that adolescents involved in multiple risk behaviors are more at risk for a number of negative outcomes, including addiction and detrimental health effects [29], reducing substance use behaviors should remain a public health priority. Health interventions aiming at multiple substance use may be initiated as early as primary school, considering the relative high prevalence of substance use experimentation among this sample of adolescents. Moreover, previous research found that the risk of dual and multiple substance use increases with age [30]; thus, intervening on substance use among young adolescents is a promising approach in reducing future substance use behaviors. In this connection, we found that alcohol use was in particular decreasing among the youngest adolescents (13-year-olds) relative to 15-year-olds—these trends may indicate a shift in alcohol use behaviors among Danish adolescents. Although school-based preventive interventions often focus on a single substance use behavior such as smoking [31], the present study indicates a wider focus on substance use prevention could be beneficial—i.e., very few adolescents smoke cigarettes without currently using alcohol. Previous research also demonstrated that use of alcohol and tobacco often cluster among adolescents [29,32].

### Methodological Considerations

An important strength of this study is the use of five comparable surveys comprising a national representative study population of Danish adolescents. Due to the school-based study design, the possibility of reaching all adolescents in the desired age groups was high. Further, the response rate was high (87%). However, some methodological considerations should be taken into account in the interpretation of results derived from the study. Although the overall response rate was high, there is still some risk of selection bias. Selection bias may either be present at the school-level, i.e., schools with specific characteristics declining to participate in the HBSC surveys. However, the risk of selection bias caused by schools’ non-participation is considered low, as most non-participating schools explained their non-participation with lack of time or that they had recently participated in a similar survey. Selection bias may also be caused by non-participating adolescents at the enrolled schools. For example, a study found that adolescents absent on the day of data collection had a higher likelihood of substance use, including tobacco, alcohol, and cannabis [33]. Thus, to some extent, this study may underestimate the magnitude of substance use among this sample of Danish adolescents. Due to the nature of this study, i.e., examining trends over time, the risk of selection bias and underreporting is less likely to be an issue.

Other methodological issues may relate to the items used to assess adolescent substance use. As we used self-reported data, there is always some risk of information bias. However, self-reported data is essential for obtaining in-depth information about adolescents’ everyday lives. Moreover, previous validation studies on adolescent smoking practices found that self-reporting of smoking was generally consistent with biochemical measures of tobacco consumption [34,35,36]. Nonetheless, there were indications that some adolescents reporting not to smoke could in fact be smoking. Consequently, there may be a risk of social desirability bias leading adolescents to underreport their substance use behaviors.

Finally, the time frame of current use of substances were rather wide, hence, including adolescents smoking cigarettes daily to more seldom than weekly as well as adolescents using alcohol daily to more seldom than monthly. Thus, the included groups comprised adolescents frequently engaging in substance use as well as more experimental users. Nonetheless, still relatively few adolescents smoke cigarettes, and few use substances in general in the youngest age groups which restrict the opportunities for dividing analyses into more substance use subgroups. Moreover, although no specific limits were given adolescents in relation to current substance use (i.e., more seldom than weekly or monthly), there may be some divergencies between alcohol and cigarette use responses due to the different question framings. This may have resulted in reduced comparability between the two measures, and future studies are encouraged to examine trends using more comparable question framings.

## 5. Conclusions

Results from the present study indicate decreasing trends in exclusive and dual current use of cigarettes and alcohol among Danish adolescents, and especially young adolescents (13-year-olds) were less likely to use either alcohol, cigarettes, or both. However, in 2018, still a little more than half of adolescents used one or two substances, and older adolescents may call for special attention in preventive efforts, since no decreasing trend in alcohol use was found among this group. Very few adolescents smoked cigarettes exclusively, and most adolescents who smoked cigarettes also used alcohol. Future preventive efforts may focus on alcohol use, dual use of cigarettes and alcohol as well as specific subgroups in high risk of using substances. Further, structural initiatives may have a substantial impact on adolescent substance use.

## Figures and Tables

**Table 1:** Characteristics of the study population at five time points, 2002–2018.

	2002	2006	2010	2014	2018
	(N = 3072)	(N = 3907)	(N = 3083)	(N = 3054)	(N = 2179)
Sociodemographic characteristics	% (n)	% (n)	% (n)	% (n)	% (n)
Gender					
Girls	50.8 (1560)	50.5 (11974)	50.0 (1541)	51.4 (1570)	48.9 (1065)
Grade					
Grade 7 (approx. 13 years)	52.7 (1619)	56.9 (2222)	53.6 (1652)	51.6 (1575)	56.1 (1223)
Grade 9 (approx. 15 years)	47.3 (1453)	43.1 (1685)	46.4 (1431)	48.4 (1479)	43.9 (956)
Country background					
Denmark	92.7 (2833)	93.3 (2870)	95.1 (2.870)	96.8 (2872)	93.9 (2031)
Other	7.3 (223)	6.7 (260)	4.9 (147)	3.2 (94)	6.2 (133)
Family occupational social class (OSC)					
High	22.6 (693)	23.5 (918)	33.4 (1029)	39.3 (1201)	36.8 (802)
Medium	50.0 (1536)	40.2 (1572)	37.6 (1158)	36.9 (1128)	37.1 (809)
Low	17.9 (551)	18.5 (723)	16.3 (503)	13.5 (413)	10.2 (222)
Non-classifiable	9.5 (292)	17.8 (694)	12.8 (393)	10.2 (312)	15.9 (346)

**Table 2:** Overall smoking and alcohol patterns at five time points, 2002–2018.

	2002	2006	2010	2014	2018	*p*-Value *
	(N = 3072)	(N = 3907)	(N = 3083)	(N = 3054)	(N = 2179)	
	% (n)	% (n)	% (n)	% (n)	% (n)	
Any current use	71.7 (2188)	64.8 (2521)	61.2 (1882)	51.7 (1572)	51.6 (1111)	<0.001
Exclusive current use of cigarettes	0.8 (25)	0.6 (24)	1.0 (30)	1.0 (30)	1.3 (27)	0.030
Exclusive current use of alcohol	52.8 (1610)	48.9 (1903)	45.4 (1395)	40.8 (1239)	41.8 (899)	<0.001
Dual current use of cigarettes and alcohol	18.1 (553)	15.3 (594)	14.9 (457)	10.0 (303)	8.6 (185)	<0.001

* test for trend, two-sided *p*-values, using Cochran-Armitage test for trend.

**Table 3:** Current substance use at five time points, 2002–2018, according to age groups.

	2002	2006	2010	2014	2018	*p*-Value *
	(N = 3072)	(N = 3907)	(N = 3083)	(N = 3054)	(N = 2179)	
	% (n)	% (n)	% (n)	% (n)	% (n)	
Any current use						
Grade 7 (approx. 13 years)	57.5 (928)	49.2 (1090)	43.1 (712)	30.5 (477)	30.7 (373)	<0.001
Grade 9 (approx. 15 years)	87.6 (1260)	85.3 (1431)	82.1 (1170)	74.2 (1095)	78.7 (738)	<0.001
Exclusive current use of cigarettes						
Grade 7 (approx. 13 years)	0.9 (14)	0.6 (13)	0.8 (13)	1.5 (23)	1.6 (19)	-
Grade 9 (approx. 15 years)	0.8 (11)	0.7 (11)	1.2 (17)	0.5 (7)	0.9 (8)	-
Exclusive current use of alcohol						
Grade 7 (approx. 13 years)	46.0 (742)	40.9 (905)	35.3 (582)	26.1 (408)	26.3 (320)	<0.001
Grade 9 (approx. 15 years)	60.4 (868)	59.5 (998)	57.1 (813)	56.3 (831)	61.7 (579)	0.493
Dual current use of cigarettes and alcohol						
Grade 7 (approx. 13 years)	10.7 (172)	7.8 (172)	7.1 (117)	2.9 (46)	2.8 (34)	<0.001
Grade 9 (approx. 15 years)	26.5 (381)	25.2 (422)	23.9 (340)	17.4 (257)	16.1 (151)	<0.001

* test for trend, two-sided *p*-values, using Cochran-Armitage test for trend. Note. due to the low number of observations in the analyses of exclusive current use of cigarettes, *p*-values have been omitted.

**Table 4:** Current substance use at five time points, 2002–2018, according to gender.

	2002	2006	2010	2014	2018	*p*-Value *
	(N = 3072)	(N = 3907)	(N = 3083)	(N = 3054)	(N = 2179)	
	% (n)	% (n)	% (n)	% (n)	% (n)	
Any current use						
Boys	72.7 (1090)	67.2 (1294)	62.3 (957)	54.0 (796)	54.0 (590)	<0.001
Girls	70.7 (1098)	62.4 (1227)	60.1 (925)	49.6 (776)	49.1 (521)	<0.001
Exclusive current use of cigarettes						
Boys	0.5 (7)	0.7 (13)	1.1 (17)	1.4 (20)	1.4 (15)	-
Girls	1.2 (18)	0.6 (11)	0.8 (13)	0.6 (10)	1.1 (12)	-
Exclusive current use of alcohol						
Boys	54.4 (815)	49.9 (962)	47.1 (724)	42.9 (632)	43.4 (474)	<0.001
Girls	51.2 (795)	47.9 (941)	43.6 (671)	38.8 (607)	40.1 (425)	<0.001
Dual current use of cigarettes and alcohol						
Boys	17.9 (268)	16.6 (319)	14.1 (216)	9.8 (144)	9.3 (101)	<0.001
Girls	18.4 (285)	14.0 (275)	15.7 (241)	10.2 (159)	7.9 (84)	<0.001

* test for trend, two-sided *p*-values, using Cochran-Armitage test for trend. Note. due to the low number of observations in the analyses of exclusive current use of cigarettes, *p*-values have been omitted.

**Table 5:** Current substance use at five time points, 2002–2018, according to gender and age groups.

		2002	2006	2010	2014	2018	*p*-Value *
		(N = 3072)	(N = 3907)	(N = 3083)	(N = 3054)	(N = 2179)	
		% (n)	% (n)	% (n)	% (n)	% (n)	
	Any current use						
Boys	Grade 7	61.2 (492)	52.2 (572)	45.8 (377)	33.3 (254)	34.3 (211)	<0.001
	Grade 9	86.0 (598)	86.8 (722)	81.2 (580)	76.2 (542)	79.6 (379)	<0.001
Girls	Grade 7	53.8 (436)	46.3 (518)	40.5 (335)	27.8 (223)	27.1 (162)	<0.001
	Grade 9	89.1 (662)	83.1 (709)	83.0 (590)	72.4 (553)	77.7 (359)	<0.001
	Exclusive current use of cigarettes						
Boys	Grade 7	0.6 (5)	0.8 (9)	0.9 (7)	2.0 (15)	1.3 (8)	-
	Grade 9	n/a	n/a	1.4 (10)	0.7 (5)	1.5 (7)	-
Girls	Grade 7	1.1 (9)	0.4 (4)	0.7 (6)	1.0 (8)	1.8 (11)	0.072
	Grade 9	1.2 (9)	0.8 (7)	1.0 (7)	n/a	n/a	n/a
	Exclusive current use of alcohol						
Boys	Grade 7	49.0 (394)	41.9 (459)	38.2 (314)	28.5 (217)	29.6 (182)	<0.001
	Grade 9	60.6 (421)	60.5 (503)	57.4 (410)	58.4 (415)	61.3 (292)	0.732
Girls	Grade 7	43.0 (348)	39.8 (446)	32.4 (268)	23.8 (191)	23.0 (138)	<0.001
	Grade 9	60.2 (447)	58.5 (495)	56.7 (403)	54.5 (416)	62.1 (287)	0.525
	Dual current use of cigarettes and alcohol						
Boys	Grade 7	11.6 (93)	9.5 (104)	6.8 (56)	2.9 (22)	3.4 (21)	<0.001
	Grade 9	25.2 (175)	25.8 (215)	22.4 (160)	17.2 (122)	16.8 (80)	<0.001
Girls	Grade 7	9.8 (79)	6.1 (68)	7.4 (61)	3.0 (24)	2.2 (13)	<0.001
	Grade 9	27.7 (206)	24.5 (207)	25.3 (180)	17.7 (135)	15.4 (71)	<0.001

* test for trend, two-sided *p*-values, using Cochran-Armitage test for trend. Note. n/a are reported in cases where cells contain observations <5. Due to the low number of observations in the analyses of exclusive current use of cigarettes, *p*-values have been omitted.

## Data Availability

Data that support the findings from this study are available. However, restrictions apply to the availability of data which were used under license for the current study and are thus, not publicly available. Data is available from the corresponding author upon reasonable request and with permission of the Danish HBSC Steering Committee.
